# Chondroblastoma-Like Chondroma of Soft Tissue Arising at the Outer Canthus: A Case Report

**DOI:** 10.1155/crop/2036386

**Published:** 2025-07-16

**Authors:** Dejan M. Rašić, Dolika D. Vasović, Jelena Sopta

**Affiliations:** ^1^Eye Hospital University Clinical Centre of Serbia, Belgrade, Serbia; ^2^Faculty of Medicine, University of Belgrade, Belgrade, Serbia; ^3^Institute of Pathology, Faculty of Medicine, University of Belgrade, Belgrade, Serbia

**Keywords:** chondroblastoma-like chondroma, histopathology, immunohistochemistry, ophthalmic soft tissue

## Abstract

Chondroblastoma-like chondroma is a rare variant of soft tissue chondroma that can mimic bone-origin chondroblastoma histologically. Its occurrence in the periocular region is extremely rare. We report a 31-year-old woman with a painless, slowly enlarging nodule at the right outer canthus. Histopathological evaluation revealed a dermal tumor composed of polygonal mononuclear cells, multinucleated giant cells, and chondroid matrix with “chicken-wire” calcifications. Immunohistochemistry supported a diagnosis of chondroblastoma-like chondroma. This case highlights the diagnostic complexity of periocular soft tissue chondromas and underscores the critical role of histopathology in achieving accurate diagnosis and informing treatment decisions.

## 1. Introduction

Chondroblastoma is a rare, benign primary bone tumor that typically arises in secondary centers of enchondral ossification [1]. According to the World Health Organization, it is characterized by highly cellular and relatively undifferentiated tissue composed of rounded or polygonal chondroblast-like cells embedded in a cartilaginous intercellular matrix [2]. Extraosseous chondroblastomas are extremely rare [3–5], and their clinical, pathological, and histogenetic features remain poorly understood, often presenting diagnostic challenges [2].

While chondroblastomas typically originate in bone, similar histological features can occasionally be observed in soft tissue lesions [3, 4]. In contrast to bone chondroblastomas, chondromas of soft tissue, also known as extraskeletal chondromas, arise independently of bone and are usually composed of mature cartilage without aggressive features. However, chondroblastoma-like chondroma represents a rare subtype of soft tissue chondroma that exhibits significant histological overlap with chondroblastoma, particularly the presence of chondroblast-like cells, osteoclast-like giant cells, immature cartilage, and characteristic “chicken-wire” calcifications [2, 4]. Despite these similarities, it arises entirely within soft tissue, without any bone involvement. This histologic mimicry underscores the importance of careful differentiation, as the clinical behavior and management may differ significantly. Proper recognition is essential for accurate diagnosis, prognostic assessment, and appropriate follow-up.

## 2. Case Report

In June 2003, a 31-year-old female presented with a gradually enlarging, painless, reddish-pink, firm nodule at the right outer canthus, which she had initially noticed a year prior. The ophthalmological examination of both eyes was unremarkable, and she was in good overall health. Clinically, the tumor appeared nonspecific, leading to straightforward excision. The patient remains well, with no evidence of local recurrence over the subsequent 20 years, with annual ophthalmological examinations during the first 5 years and biannual reviews thereafter. Written consent was obtained from the patient for the inclusion of her case details.

### 2.1. Ocular Pathology

**Figure 1:**
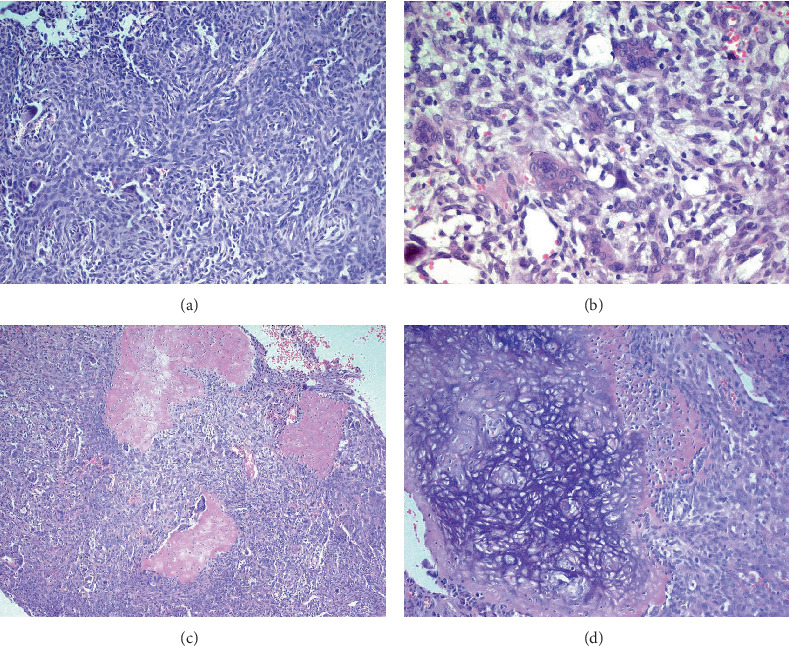
(a) Light microscopy shows highly cellular sheets of neoplastic polygonal to elongated mononuclear cells with eosinophilic cytoplasm, pale vesicular nuclei, and indistinct borders. (b) Deeper layers exhibit spindle cells, osteoclast-like giant cells, and endothelial-lined vessels, some containing multinucleated giant cells. (c, d) The pink chondroid matrix, hemorrhagic cystic areas, and cartilage foci with “chicken-wire” calcifications are clearly visible. Chondroblast nuclei show hyperchromatic features with longitudinal grooves.

The excised specimen, a whitish-gray-brown, firm tissue, was fixed in 4% neutral buffered formalin and submitted for routine histopathological examination. It measured 4 × 3 × 2 mm. The specimen was paraffin-embedded, sectioned at 5 *μ*m, and stained with H&E, followed by immunohistochemical analysis. Light microscopic examination revealed a distinct, nonencapsulated, and highly cellular dermal tumor composed of sheets of neoplastic polygonal to elongated mononuclear cells with abundant eosinophilic cytoplasm, pale vesicular nuclei with nucleoli, and indistinct borders (Figure 1a). In deeper layers, spindle cells resembling fibroblasts with mild cellular atypia and osteoclast-like giant cells with 5–20 nuclei were observed (Figure 1b). Focal areas of spindle cells exhibited storiform-like patterns. Vascular structures within the tumor displayed a range of morphologies, from endothelial lining with branching slit-like shapes to occasional connections with multinucleated giant cells (Figure 1c). The intercellular matrix showed a chondroid composition with a pinkish hue (Figure 1d). Hemorrhagic cystic areas lacking endothelial lining were prevalent and tended to coalesce. Juxtaposed mononuclear cell proliferation areas contained foci of both immature and mature cartilage (Figure 1c). Linear calcifications outlining the lacunae of individual chondroblasts formed a distinctive “chicken-wire” or “lace-like” pattern. The hyperchromatic nuclei of chondroblasts displayed characteristic longitudinal grooves.

### 2.2. Immunohistochemical Findings

**Figure 2:**
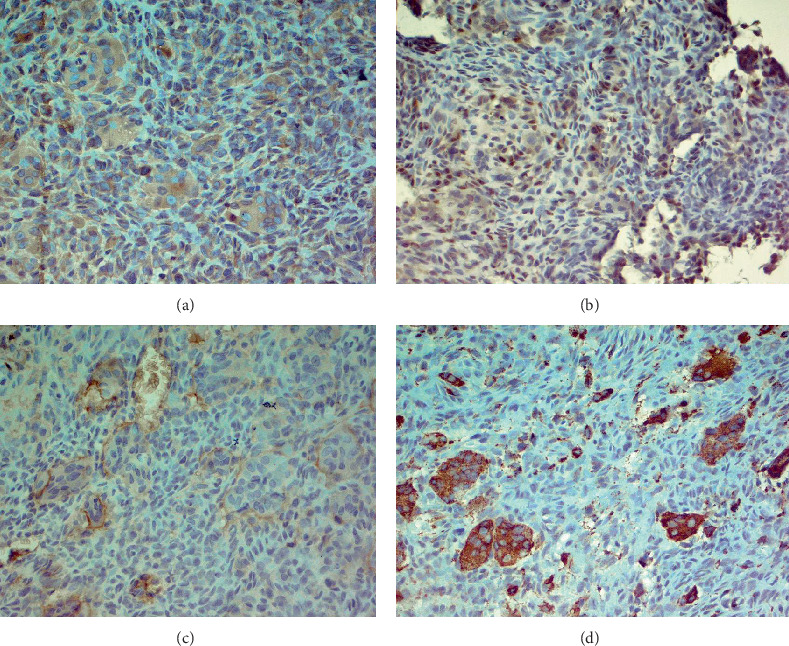
Tumor cells show positivity for vimentin and S-100 antibodies, with nuclear, perinuclear, and weak diffuse cytoplasmic staining; (a, b) giant cells exhibit well-defined cytoplasmic positivity. (c, d) Multinucleated giant cells are positive for leukocyte common antigen and show strong granular cytoplasmic positivity for CD68.

Immunohistochemically, most tumor cells were positive for vimentin and S-100 antibodies, displaying nuclear, perinuclear, and weak diffuse cytoplasmic staining. Giant cells demonstrated well-defined cytoplasmic positivity (Figure 2a,b). Multinucleated giant cells stained positive for antibodies against leucocyte common antigen (LCA) (membrane positivity, which delineates the rather indistinct cellular borders of these cells as seen in H&E-stained specimens) (Figure 2c). Multinucleated giant cells were also strongly positive for antibodies against CD68 (granular cytoplasmic positivity) (Figure 2d).

### 2.3. Differential Diagnoses

Differential diagnoses included cutaneous giant cell fibroma with chondroid differentiation, localized giant cell tumor of tendon sheath, fibroma of tendon sheath, deep benign fibrous histiocytoma, aneurysmal (angiomatoid) fibrous histiocytoma, epithelioid benign fibrous histiocytoma, multinucleate cell angiohistiocytoma, giant cell fibroblastoma, calcifying aponeurotic fibroma, giant cell tumor of soft tissue, giant cell angiofibroma, cellular angiofibroma, giant cell reparative granuloma, benign giant cell tumor of bone, aneurysmal bone cyst, and metastatic chondroblastoma.

## 3. Discussion

Soft-tissue chondromas, also referred to as extraskeletal chondromas or chondromas of soft parts, are uncommon benign cartilage-forming tumors. They predominantly arise in areas adjacent to tendons, particularly within the tenosynovial sheets or soft tissues [1]. These tumors are commonly found in the region of the fingers but can occur elsewhere, including the eyelids. The World Health Organization prefers the term “soft tissue chondroma” to describe these lesions [1, 2].

Histologically, soft-tissue chondromas can present with features that make their appearance complex and potentially confusing. These features often resemble those of chondroblastoma of bone, characterized by hypercellularity, the presence of interspersed osteoclast-like multinucleated giant cells, and a sparse chondroid matrix. This matrix typically appears slightly eosinophilic and primitive, displaying delicate “lace-like” or “chicken-wire” calcifications that outline the chondrocyte lacunae. This resemblance creates significant diagnostic challenges in distinguishing soft tissue chondromas from bone chondroblastomas.

In the literature, various reports illustrate the diverse manifestations of soft tissue chondromas in the periocular region. For instance, a case initially reported as a cartilaginous choristoma of the eyelid tarsus by Mauriello et al. likely represented a soft tissue chondroma based on its microscopic description [6]. Additional cases documented by Aseem et al. and AlHazzani et al. provide further clinical context for eyelid-based chondromas, typically characterized by mature cartilage and minimal cellular atypia [7, 8]. Although rare, orbital chondromas have also been described in studies by Harrison et al., Kabra et al., and Choi et al., highlighting variations in presentation and surgical management [9–11].

Notably, the previously reported periocular cases lacked the aggressive histologic features observed in our patient. Unlike these typical chondromas, our case exhibited high cellularity, the presence of both immature and mature cartilage, osteoclast-like giant cells, and distinctive “chicken-wire” calcifications—features more consistent with chondroblastoma. These distinctions underscore the importance of recognizing chondroblastoma-like chondroma as a distinct pathological entity within the differential diagnosis of periocular cartilage-forming tumors.

## 4. Conclusion

The histopathological features of our case—including the tumor's mesenchymal origin, chondroid ground substance, mononuclear neoplastic cells in various stages of maturation toward chondroblasts, numerous osteoclast-like multinucleated giant cells, areas of immature and mature cartilage with “chicken-wire” calcifications, and extensive intratumoral hemorrhage—support the diagnosis of a chondroblastoma-like chondroma of soft tissue in the eyelid. To the best of our knowledge, this is the first described case of chondroblastoma-like chondroma of soft tissue in the periocular region.

## Data Availability

The data that support the findings of this study are available from the corresponding author upon reasonable request.
